# Ethyl 1-(2,6-difluoro­benz­yl)-1*H*-1,2,3-triazole-4-carboxyl­ate

**DOI:** 10.1107/S1600536810050014

**Published:** 2010-12-15

**Authors:** Jing Jia, Dingqiang Lu

**Affiliations:** a School of Pharmaceutical Sciences, Nanjing University of Technology, No. 5 Xinmofan Road, Nanjing 210009, People’s Republic of China and Jiangsu Provincial Institute of Materia Medica, Nanjing University of Technology, No. 26 Majia Street, Nanjing 210009, People’s Republic of China

## Abstract

In the title compound, C_12_H_11_F_2_N_3_O_2_, the dihedral angle between the triazole and phenyl rings is 73.74 (9)°. In the crystal, mol­ecules are linked into chains along [010] *via* weak C—H⋯O and C—H⋯N hydrogen bonds.

## Related literature

The title compound is an inter­mediate in the synthesis of rufinamide, a new anti-epilepsy drug (Herranz, 2008 ▶). For synthetic procedures, see: Abu-Orabi *et al.* (1989 ▶); Wang & Xie (2004 ▶). For a related structure, see: Xiao *et al.* (2008 ▶).
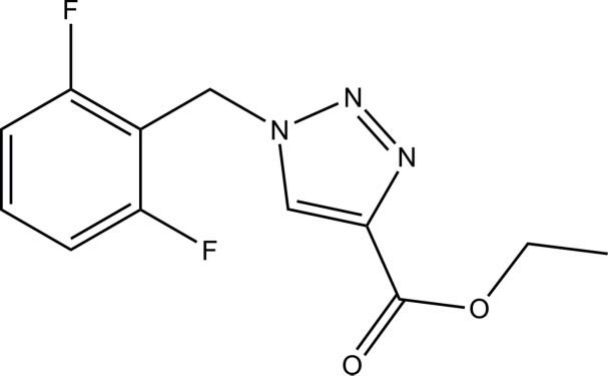


## Experimental

### 

#### Crystal data

C_12_H_11_F_2_N_3_O_2_
*M* *_r_* = 267.24
Monoclinic, 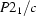

*a* = 9.4540 (19) Å
*b* = 10.963 (2) Å
*c* = 12.167 (2) Å
β = 93.21 (3)°
*V* = 1259.1 (4) Å^3^
*Z* = 4
Mo *K*α radiation
μ = 0.12 mm^−1^
*T* = 293 K
0.30 × 0.20 × 0.20 mm

#### Data collection

Enraf–Nonius CAD-4 diffractometer
Absorption correction: ψ scan (North *et al.*, 1968 ▶) *T*_min_ = 0.965, *T*_max_ = 0.977
3270 measured reflections
2316 independent reflections
1629 reflections with *I* > 2σ(*I*)
*R*_int_ = 0.026
3 standard reflections every 200 reflections  intensity decay: 1%

#### Refinement

*R*[*F* ^2^ > 2σ(*F* ^2^)] = 0.055
*wR*(*F* ^2^) = 0.163
*S* = 1.03
2316 reflections
174 parameters
H-atom parameters constrained
Δρ_max_ = 0.32 e Å^−3^
Δρ_min_ = −0.22 e Å^−3^

### 

Data collection: *CAD-4 EXPRESS* (Enraf–Nonius, 1994 ▶); cell refinement: *CAD-4 EXPRESS*; data reduction: *XCAD4* (Harms & Wocadlo, 1995 ▶); program(s) used to solve structure: *SHELXS97* (Sheldrick, 2008 ▶); program(s) used to refine structure: *SHELXL97* (Sheldrick, 2008 ▶); molecular graphics: *SHELXTL* (Sheldrick, 2008 ▶); software used to prepare material for publication: *PLATON* (Spek, 2009 ▶).

## Supplementary Material

Crystal structure: contains datablocks global, I. DOI: 10.1107/S1600536810050014/pv2364sup1.cif

Structure factors: contains datablocks I. DOI: 10.1107/S1600536810050014/pv2364Isup2.hkl

Additional supplementary materials:  crystallographic information; 3D view; checkCIF report

## Figures and Tables

**Table 1:** Hydrogen-bond geometry (Å, °)

*D*—H⋯*A*	*D*—H	H⋯*A*	*D*⋯*A*	*D*—H⋯*A*
C7—H7*B*⋯O1^i^	0.97	2.47	3.415 (3)	166
C8—H8⋯N3^i^	0.93	2.61	3.536 (3)	172

Symmetry code: (i) 
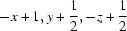
.

**Fig. 1.**
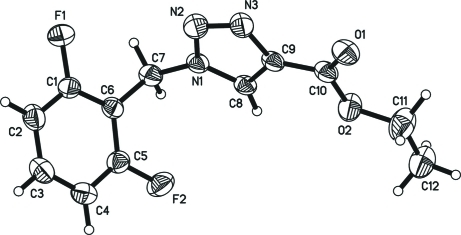
ORTEP view of the title compound. The dispalcement ellipsoids are drawn at 30% probability level.

**Fig. 2.**
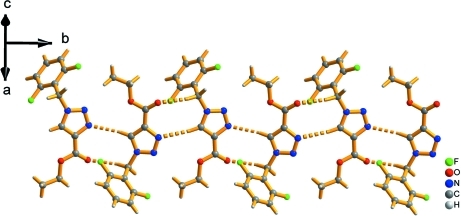
A one-dimensional chain of the title compound.

## supplementary crystallographic information

### Comment

Epilepsia has been a common disease for a long time and has been on an increase year after year. Rufinamide is a new drug to cure epilepsia; it is a triazole derivative (Herranz *et al.*, 2008). We report herein the crystal structure of the title compound which is a key intermediate in the synthesis of rufinamide. In the title compound (Fig. 1), the planes of the triazole and phenyl rings are not coplanar [dihedral angle 73.74 (9)°]. The discrete molecules are linked through weak C—H···O and C—H···N hydrogen bonds, forming one-dimensional chains along [010] direction (Fig. 2 and Tab. 1). In the structure of the title compound, the bond lengths and angles agree with the corresponding values reported for a related compound (Xiao *et al.*, 2008).

### Experimental

The title compound, was prepared by following procedures reported earlier (Wang *et al.*, 2004; Abu-Orabi *et al.*, 1989). To a solution of 2-(azidomethyl)-1,3-difluorobenzene (1.69 g, 10 mmol) in etanol (50 mL), ethyl propiolate (0.98 g, 10 mmol) was added and the mixture was heated under reflux for 10 h. After removing the solvent under reduced pressure the residue was dissolved and the title compound recrystallized from petroleum ether-methanol mixture (15:2), to provide crystals suitable for X-ray diffraction (yield 2.31 g, 86.3%).

### Refinement

H atoms were palced in geometrically calculated position and were refined using a riding model, with C—H = 0.93, 0.96 and 0.97 Å, for aryl, methyl and methylene type H-atoms, respectively, and *U*_iso_(H) = 1.5 and 1.2 *U*_eq_(C) for methyl and nonmethyl H-atoms, respectively.

### Crystal data

**Table:**

C_12_H_11_F_2_N_3_O_2_	*F*(000) = 552
*M**_r_* = 267.24	*D*_x_ = 1.410 Mg m^−^^3^
Monoclinic, *P*2_1_/*c*	Mo *K*α radiation, λ = 0.71073 Å
Hall symbol: -P 2ybc	Cell parameters from 25 reflections
*a* = 9.4540 (19) Å	θ = 9–13°
*b* = 10.963 (2) Å	µ = 0.12 mm^−^^1^
*c* = 12.167 (2) Å	*T* = 293 K
β = 93.21 (3)°	Block, colorless
*V* = 1259.1 (4) Å^3^	0.30 × 0.20 × 0.20 mm
*Z* = 4	

### Data collection

**Table:**

Enraf–Nonius CAD-4 diffractometer	1629 reflections with *I* > 2σ(*I*)
Radiation source: fine-focus sealed tube	*R*_int_ = 0.026
graphite	θ_max_ = 25.4°, θ_min_ = 2.2°
ω/2θ scans	*h* = 0→11
Absorption correction: ψ scan (North *et al.*, 1968)	*k* = −3→13
*T*_min_ = 0.965, *T*_max_ = 0.977	*l* = −14→14
3270 measured reflections	3 standard reflections every 200 reflections
2316 independent reflections	intensity decay: 1%

### Refinement

**Table:**

Refinement on *F*^2^	Secondary atom site location: difference Fourier map
Least-squares matrix: full	Hydrogen site location: inferred from neighbouring sites
*R*[*F*^2^ > 2σ(*F*^2^)] = 0.055	H-atom parameters constrained
*wR*(*F*^2^) = 0.163	*w* = 1/[σ^2^(*F*_o_^2^) + (0.0873*P*)^2^ + 0.337*P*] where *P* = (*F*_o_^2^ + 2*F*_c_^2^)/3
*S* = 1.03	(Δ/σ)_max_ < 0.001
2316 reflections	Δρ_max_ = 0.32 e Å^−^^3^
174 parameters	Δρ_min_ = −0.22 e Å^−^^3^
0 restraints	Extinction correction: *SHELXL97* (Sheldrick, 2008), Fc^*^=kFc[1+0.001xFc^2^λ^3^/sin(2θ)]^-1/4^
Primary atom site location: structure-invariant direct methods	Extinction coefficient: 0.029 (5)

### Special details

**Table:**

Geometry. All e.s.d.'s (except the e.s.d. in the dihedral angle between two l.s. planes) are estimated using the full covariance matrix. The cell e.s.d.'s are taken into account individually in the estimation of e.s.d.'s in distances, angles and torsion angles; correlations between e.s.d.'s in cell parameters are only used when they are defined by crystal symmetry. An approximate (isotropic) treatment of cell e.s.d.'s is used for estimating e.s.d.'s involving l.s. planes.
Refinement. Refinement of *F*^2^ against ALL reflections. The weighted *R*-factor *wR* and goodness of fit *S* are based on *F*^2^, conventional *R*-factors *R* are based on *F*, with *F* set to zero for negative *F*^2^. The threshold expression of *F*^2^ > σ(*F*^2^) is used only for calculating *R*-factors(gt) *etc*. and is not relevant to the choice of reflections for refinement. *R*-factors based on *F*^2^ are statistically about twice as large as those based on *F*, and *R*- factors based on ALL data will be even larger.

### Fractional atomic coordinates and isotropic or equivalent isotropic displacement parameters (Å^2^)

**Table:**

	*x*	*y*	*z*	*U*_iso_*/*U*_eq_	
F1	0.94152 (18)	0.27880 (14)	0.02828 (16)	0.0890 (6)	
F2	0.78127 (17)	0.61613 (15)	0.22000 (14)	0.0829 (6)	
O1	0.4165 (2)	0.20776 (17)	0.46337 (16)	0.0744 (6)	
O2	0.3594 (2)	0.39959 (17)	0.41799 (17)	0.0776 (6)	
N1	0.62925 (19)	0.35347 (16)	0.16945 (17)	0.0537 (5)	
N2	0.6558 (3)	0.2343 (2)	0.1898 (2)	0.0751 (7)	
N3	0.5829 (2)	0.20178 (19)	0.2728 (2)	0.0709 (7)	
C1	0.9678 (3)	0.3771 (2)	0.0933 (2)	0.0618 (7)	
C2	1.1060 (3)	0.4031 (3)	0.1262 (3)	0.0733 (8)	
H2	1.1796	0.3538	0.1049	0.088*	
C3	1.1325 (3)	0.5034 (3)	0.1910 (3)	0.0751 (8)	
H3	1.2252	0.5220	0.2146	0.090*	
C4	1.0243 (3)	0.5767 (3)	0.2217 (2)	0.0687 (7)	
H4	1.0426	0.6459	0.2644	0.082*	
C5	0.8887 (3)	0.5456 (2)	0.1878 (2)	0.0583 (6)	
C6	0.8534 (2)	0.4459 (2)	0.12140 (19)	0.0522 (6)	
C7	0.7032 (3)	0.4176 (2)	0.0843 (2)	0.0593 (7)	
H7B	0.6538	0.4930	0.0658	0.071*	
H7A	0.7023	0.3676	0.0185	0.071*	
C8	0.5379 (2)	0.3966 (2)	0.2397 (2)	0.0524 (6)	
H8	0.5015	0.4753	0.2428	0.063*	
C9	0.5093 (2)	0.3001 (2)	0.3059 (2)	0.0538 (6)	
C10	0.4248 (3)	0.2941 (2)	0.4032 (2)	0.0577 (6)	
C11	0.2781 (4)	0.4100 (3)	0.5169 (3)	0.1053 (13)	
H11A	0.2056	0.3473	0.5162	0.126*	
H11B	0.3404	0.3988	0.5822	0.126*	
C12	0.2137 (4)	0.5286 (3)	0.5186 (3)	0.0955 (11)	
H12A	0.1482	0.5373	0.4559	0.143*	
H12C	0.2857	0.5901	0.5163	0.143*	
H12B	0.1641	0.5376	0.5849	0.143*	

### Atomic displacement parameters (Å^2^)

**Table:**

	*U*^11^	*U*^22^	*U*^33^	*U*^12^	*U*^13^	*U*^23^
F1	0.0929 (12)	0.0560 (10)	0.1202 (14)	−0.0053 (8)	0.0250 (10)	−0.0258 (9)
F2	0.0797 (11)	0.0754 (11)	0.0936 (12)	0.0159 (9)	0.0056 (9)	−0.0255 (9)
O1	0.0857 (13)	0.0520 (11)	0.0853 (13)	−0.0042 (9)	0.0038 (10)	0.0175 (10)
O2	0.0897 (14)	0.0537 (11)	0.0929 (14)	0.0125 (10)	0.0351 (11)	0.0150 (10)
N1	0.0500 (11)	0.0360 (10)	0.0751 (13)	−0.0006 (8)	0.0039 (9)	−0.0005 (9)
N2	0.0744 (15)	0.0421 (12)	0.1107 (18)	0.0101 (11)	0.0221 (14)	0.0037 (12)
N3	0.0678 (14)	0.0404 (12)	0.1064 (18)	0.0058 (10)	0.0220 (13)	0.0086 (12)
C1	0.0709 (17)	0.0402 (12)	0.0759 (16)	−0.0045 (12)	0.0198 (13)	−0.0027 (11)
C2	0.0581 (16)	0.0583 (17)	0.105 (2)	0.0027 (13)	0.0198 (15)	0.0076 (15)
C3	0.0602 (16)	0.0719 (19)	0.093 (2)	−0.0104 (15)	0.0015 (15)	0.0048 (16)
C4	0.0718 (17)	0.0629 (16)	0.0706 (17)	−0.0111 (14)	−0.0030 (13)	−0.0067 (13)
C5	0.0621 (15)	0.0524 (14)	0.0607 (14)	0.0023 (12)	0.0061 (12)	−0.0008 (11)
C6	0.0562 (13)	0.0444 (12)	0.0563 (13)	−0.0040 (11)	0.0066 (10)	0.0055 (10)
C7	0.0614 (15)	0.0506 (14)	0.0658 (15)	−0.0042 (11)	0.0018 (12)	−0.0001 (12)
C8	0.0439 (12)	0.0359 (12)	0.0769 (16)	0.0018 (9)	−0.0008 (11)	−0.0022 (11)
C9	0.0460 (12)	0.0375 (12)	0.0775 (16)	−0.0028 (10)	−0.0004 (11)	0.0011 (11)
C10	0.0534 (13)	0.0434 (13)	0.0759 (16)	−0.0046 (11)	−0.0003 (12)	0.0039 (12)
C11	0.141 (3)	0.077 (2)	0.103 (3)	0.020 (2)	0.058 (2)	0.0216 (19)
C12	0.105 (2)	0.095 (3)	0.088 (2)	0.018 (2)	0.0283 (18)	0.0080 (19)

### Geometric parameters (Å, °)

**Table:**

F1—C1	1.352 (3)	C4—C5	1.368 (4)
F2—C5	1.352 (3)	C4—H4	0.9300
O1—C10	1.202 (3)	C5—C6	1.390 (3)
O2—C10	1.329 (3)	C6—C7	1.498 (3)
O2—C11	1.468 (4)	C7—H7B	0.9700
N1—C8	1.335 (3)	C7—H7A	0.9700
N1—N2	1.351 (3)	C8—C9	1.366 (3)
N1—C7	1.462 (3)	C8—H8	0.9300
N2—N3	1.304 (3)	C9—C10	1.466 (4)
N3—C9	1.356 (3)	C11—C12	1.437 (4)
C1—C2	1.374 (4)	C11—H11A	0.9700
C1—C6	1.377 (3)	C11—H11B	0.9700
C2—C3	1.368 (4)	C12—H12A	0.9600
C2—H2	0.9300	C12—H12C	0.9600
C3—C4	1.369 (4)	C12—H12B	0.9600
C3—H3	0.9300		
			
C10—O2—C11	116.6 (2)	C6—C7—H7B	109.3
C8—N1—N2	110.2 (2)	N1—C7—H7A	109.3
C8—N1—C7	129.4 (2)	C6—C7—H7A	109.3
N2—N1—C7	120.2 (2)	H7B—C7—H7A	108.0
N3—N2—N1	107.8 (2)	N1—C8—C9	105.03 (19)
N2—N3—C9	108.3 (2)	N1—C8—H8	127.5
F1—C1—C2	118.4 (2)	C9—C8—H8	127.5
F1—C1—C6	117.4 (2)	N3—C9—C8	108.6 (2)
C2—C1—C6	124.2 (2)	N3—C9—C10	121.0 (2)
C3—C2—C1	118.3 (3)	C8—C9—C10	130.2 (2)
C3—C2—H2	120.8	O1—C10—O2	123.7 (2)
C1—C2—H2	120.8	O1—C10—C9	125.8 (2)
C2—C3—C4	120.9 (3)	O2—C10—C9	110.4 (2)
C2—C3—H3	119.5	C12—C11—O2	108.9 (3)
C4—C3—H3	119.5	C12—C11—H11A	109.9
C5—C4—C3	118.2 (3)	O2—C11—H11A	109.9
C5—C4—H4	120.9	C12—C11—H11B	109.9
C3—C4—H4	120.9	O2—C11—H11B	109.9
F2—C5—C4	118.5 (2)	H11A—C11—H11B	108.3
F2—C5—C6	117.3 (2)	C11—C12—H12A	109.5
C4—C5—C6	124.2 (2)	C11—C12—H12C	109.5
C1—C6—C5	114.1 (2)	H12A—C12—H12C	109.5
C1—C6—C7	123.8 (2)	C11—C12—H12B	109.5
C5—C6—C7	122.0 (2)	H12A—C12—H12B	109.5
N1—C7—C6	111.7 (2)	H12C—C12—H12B	109.5
N1—C7—H7B	109.3		
			
C8—N1—N2—N3	−0.5 (3)	C8—N1—C7—C6	100.9 (3)
C7—N1—N2—N3	176.4 (2)	N2—N1—C7—C6	−75.2 (3)
N1—N2—N3—C9	0.0 (3)	C1—C6—C7—N1	99.0 (3)
F1—C1—C2—C3	179.3 (3)	C5—C6—C7—N1	−81.2 (3)
C6—C1—C2—C3	0.1 (4)	N2—N1—C8—C9	0.7 (3)
C1—C2—C3—C4	−0.6 (4)	C7—N1—C8—C9	−175.8 (2)
C2—C3—C4—C5	1.4 (4)	N2—N3—C9—C8	0.4 (3)
C3—C4—C5—F2	178.5 (3)	N2—N3—C9—C10	−175.6 (2)
C3—C4—C5—C6	−1.9 (4)	N1—C8—C9—N3	−0.7 (3)
F1—C1—C6—C5	−179.7 (2)	N1—C8—C9—C10	174.9 (2)
C2—C1—C6—C5	−0.5 (4)	C11—O2—C10—O1	2.7 (4)
F1—C1—C6—C7	0.1 (4)	C11—O2—C10—C9	−176.0 (3)
C2—C1—C6—C7	179.4 (2)	N3—C9—C10—O1	3.0 (4)
F2—C5—C6—C1	−179.0 (2)	C8—C9—C10—O1	−172.1 (3)
C4—C5—C6—C1	1.4 (4)	N3—C9—C10—O2	−178.3 (2)
F2—C5—C6—C7	1.2 (3)	C8—C9—C10—O2	6.6 (4)
C4—C5—C6—C7	−178.4 (2)	C10—O2—C11—C12	−179.3 (3)

### Hydrogen-bond geometry (Å, °)

**Table:**

*D*—H···*A*	*D*—H	H···*A*	*D*···*A*	*D*—H···*A*
C7—H7A···F1	0.97	2.46	2.833 (3)	103.
C7—H7B···O1^i^	0.97	2.47	3.415 (3)	166.
C8—H8···N3^i^	0.93	2.61	3.536 (3)	172.

Symmetry codes: (i) −*x*+1, *y*+1/2, −*z*+1/2.
